# PI-YOLO: dynamic sparse attention and lightweight convolutional based YOLO for vessel detection in pathological images

**DOI:** 10.3389/fonc.2024.1347123

**Published:** 2024-07-29

**Authors:** Cong Li, Shuanlong Che, Haotian Gong, Youde Ding, Yizhou Luo, Jianing Xi, Ling Qi, Guiying Zhang

**Affiliations:** ^1^ The Affiliated Qingyuan Hospital (Qingyuan Peoples’s Hospital), Guangzhou Medical University, Qingyuan, China; ^2^ School of Biomedical Engineering, Guangzhou Medical University, Guangzhou, China; ^3^ Department of Pathology, Guangzhou KingMed Center for Clinical Laboratory, Guangzhou, China; ^4^ School of Health Management, Guangzhou Medical University, Guangzhou, China; ^5^ Division of Gastroenterology, Institute of Digestive Disease, the Affiliated Qingyuan Hospital (Qingyuan Peoples’s Hospital), Guangzhou Medical University, Qingyuan, China

**Keywords:** pathological images, blood vessel, deep learning, object detection, attention mechanism

## Abstract

Vessel density within tumor tissues strongly correlates with tumor proliferation and serves as a critical marker for tumor grading. Recognition of vessel density by pathologists is subject to a strong inter-rater bias, thus limiting its prognostic value. There are many challenges in the task of object detection in pathological images, including complex image backgrounds, dense distribution of small targets, and insignificant differences between the features of the target to be detected and the image background. To address these problems and thus help physicians quantify blood vessels in pathology images, we propose Pathological Images-YOLO (PI-YOLO), an enhanced detection network based on YOLOv7. PI-YOLO incorporates the BiFormer attention mechanism, enhancing global feature extraction and accelerating processing for regions with subtle differences. Additionally, it introduces the CARAFE upsampling module, which optimizes feature utilization and information retention for small targets. Furthermore, the GSConv module improves the ELAN module, reducing model parameters and enhancing inference speed while preserving detection accuracy. Experimental results show that our proposed PI-YOLO network has higher detection accuracy compared to Faster-RCNN, SSD, RetinaNet, YOLOv5 network, and the latest YOLOv7 network, with a mAP value of 87.48%, which is 2.83% higher than the original model. We also validated the performance of this network on the ICPR 2012 mitotic dataset with an F1 value of 0.8678, outperforming other methods, demonstrating the advantages of our network in the task of target detection in complex pathology images.

## 1 Introduction

The growth of most tumors is highly correlated with new blood vessels (1). Rapid tumor cell proliferation often results in hypoxia and malnutrition, prompting the formation of new blood vessels to fulfill the increased metabolic demands of tumors (2). According to the tumor angiogenesis switch hypothesis, when tumors grow to a diameter of 1-2 mm, they frequently trigger the expression of angiogenesis-related factors, initiating the formation of a new vascular network that promotes tumor growth and development. Blocking angiogenesis and tumor growth is an effective approach to treating tumors, such as colorectal cancer, lung cancer, and breast cancer (3). Further studies have revealed that quantitative analysis of blood vessels in tumors can help physicians determine tumor grade and predict patient prognosis (4). This, in turn, supports the development of more rational and effective treatment strategies. Therefore, there is a pressing need for a rapid and precise method to detect blood vessels within tumors.

In the past, there were three main methods for detecting blood vessels within tumors. However, none of these methods employed computer-based automatic detection due to issues such as limitations in imaging equipment. The first method involves the utilization of immunohistochemistry technology to selectively label specific antibodies targeting vascular endothelial cells, such as F8-RA, CD31, CD34, CD105 (5). Researchers then count the positive cells per unit area under a microscope. This method is one of the earliest approaches used for quantitative analysis of tumor vasculature and currently stands as the gold standard for such analysis (6). However, it requires manual selection of the area with the highest vessel density for counting, making it susceptible to subjective influences. The second method entails the use of target-enhanced ultrasound imaging of molecular markers that are overexpressed during angiogenesis (7), enabling indirect quantitative analysis of blood vessels. This approach has advantages such as low detection costs and real-time imaging capabilities but is limited by low detection sensitivity and limited penetration. The third method involves the targeted introduction of magnetic contrast agents into the tumor region, followed by high-resolution imaging of blood vessels within the tumor using MRI technology (8). This method, while capable of producing detailed images, demands sophisticated equipment and longer imaging times, thus limiting its clinical applicability.

In recent years, the field of histopathology has achieved significant advances through electron microscopic imaging, enabling pathologists to perform high-resolution tumor vascularization through digitized whole slide images (WSIs) (9). In addition, rapid advances in artificial intelligence technologies, particularly deep learning, have provided powerful tools for automated tissue section analysis, promising to provide more accurate and consistent results than traditional manual evaluations and to reduce the workload of pathologists. Artificial intelligence algorithms have been developed to identify and quantify vascular features such as density, morphology, and spatial distribution, which are often challenging for human observers (10). Studies have demonstrated the feasibility and efficacy of AI for vascular detection in histological sections of a wide range of malignancies, helping to improve the accuracy of lymphovascular invasion detection, predict lymph node metastasis, and identify new morphological features with prognostic value (11). However, implementing AI-based vascular testing in clinical practice still faces a number of challenges, including the need for larger and more diverse datasets, and optimizing algorithms for better and faster testing performance so that testing models can be integrated with existing pathology workflows (12).

In response to challenges posed by small target proportions, complex image backgrounds, and subtle feature differences in pathology images, we propose a YOLOv7-based detection network for object detection in pathology images (13). Our approach also prioritizes meeting the speed requirements of clinical applications. The model fuses the BiFormer (14) attention mechanism, the lightweight generalized upsampling operator CARAFE (15) and a new lightweight convolutional technique GSConv (16) into the YOLOv7 model. The proposed model significantly enhances the accuracy of blood vessel detection in pathology images and offers an effective solution for target detection in pathology images.

The contributions of this paper are as follows:

1. This article proposes an improved object detection network model for pathological images based on YOLOv7. We fused the BiFormer attention mechanism, the CARAFE upsampling operator, and GSConv into the YOLOv7 model. This fusion concept effectively enhances detection accuracy and accelerates the blood vessel detection process in pathology images, offering an efficient solution for the task of target detection in pathology images.

2. On the Blood vessel detection dataset, PI-YOLO achieves a mean Average Precision (mAP) value of 87.48%, which is 2.83% higher than the original model. On the ICPR2012 Mitosis detection dataset, the F1 score reaches 0.8678. PI-YOLO outperforms other methods on both datasets, demonstrating superior detection accuracy and faster inference speed (17).

3. Extensive comparative and ablation experiments have provided both quantitative and qualitative verification of this model’s superiority in vascular detection tasks within pathological images from various perspectives. The outcomes of this study are anticipated to be valuable for researchers in the fields of anti-angiogenic therapy for tumors and tumor prognosis prediction.

## 2 Related work

At present, classical object detection networks can be broadly categorized into two groups: anchor-based and anchor-free. The key distinction lies in the fact that anchor-based methods require the prior definition of anchor boxes, whereas anchor-free methods do not necessitate this step. One-stage anchor-based approaches, exemplified by YOLOv3 (18) and RetinaNet (19), are capable of directly performing regression and classification tasks for bounding boxes. These methods produce outputs in the form of regression parameters (anchor offsets) and category confidences. On the other hand, the mainstream two-stage anchor-based methods, such as Faster RCNN (20) and Mask RCNN (21), initially generate proposals and subsequently conduct regression and classification tasks for the bounding boxes. Similarly, a variety of anchor-free techniques have been developed, including CornerNet (22) and FSAF (23). Among these, CornerNet is a classic example of the keypoint detection network, while FSAF incorporates a feature selection anchor-free module to achieve anchor-free object detection. While these conventional networks have delivered promising results in the context of natural images, their performance will be constrained when applied to the unique characteristics of pathological images during the detection process.

Pathological diagnosis, as the gold standard for cancer diagnosis, provides comprehensive information about tumors. In recent years, deep learning methods have been widely applied in the detection and segmentation of micro vessels in pathological images. Traditional methods rely on immunohistochemistry (IHC) staining and manual counting, which are not only time-consuming and labor-intensive but also highly subjective. To address these issues, Yi et al. (24) developed an automated detection method based on fully convolutional networks (FCNs). This method leverages deep learning to achieve end-to-end image training and pixel-level prediction, significantly improving detection efficiency. However, limitations such as small dataset sizes and high false-positive rates remain significant drawbacks. To further enhance detection accuracy and reliability, Fraz et al. (25) proposed a method for micro vessel segmentation in H&E-stained histological images. This method incorporates an uncertainty prediction mechanism that generates uncertainty maps by introducing random transformations during testing, highlighting areas where the network’s predictions are uncertain, thus improving segmentation confidence. Additionally, they developed a novel Feature Attention-Based Network (FABnet) (26) for the simultaneous segmentation of micro vessels and nerves. FABnet combines feature attention modules and uncertainty prediction mechanisms to focus on salient features and perform multi-scale feature extraction, achieving more precise segmentation. Despite significant progress in accuracy and reliability, the complexity of the network architecture and the need for multiple random transformations increase computational costs. Furthermore, the study primarily focuses on oral squamous cell carcinoma datasets, lacking extensive validation across other cancer types. Additionally, Generative Adversarial Networks (GANs) have been introduced into vascular detection. Atzori et al. (27) employed GANs to generate synthetic ERG-stained images, reducing dependency on IHC staining. Although GANs have shown impressive results in improving image quality and accuracy, issues such as variability in staining quality and limited training dataset sizes persist. All these methods are based on segmentation approaches, which involve pixel-level classification to distinguish blood vessel boundaries from the background. While accuracy has been continuously improving, the complexity of these models often results in slower processing speeds, limiting their clinical practicality.

## 3 Materials and methods

### 3.1 Datasets

In this paper, two datasets are used for experiments. The experiments on blood vessel detection in pathology images were performed on the blood vessel detection dataset we created, and the comparison experiments on other detection tasks were performed on the ICPR 2012 mitosis detection dataset (17).

#### 3.1.1 Blood vessel detection dataset

Blood vessels exist in different tumor tissues. To make the detection model applicable to various tumor tissues, we collect 36 WSIs from two institutions: TCGA (28) and the Guangzhou Kingmed Center for Clinical Laboratory. The dataset comprises twenty WSIs of breast cancer, eight WSIs of lung cancer, and eight WSIs of colon cancer. Due to the typically large size of WSIs, we divide them into patches measuring 512 × 512 pixels to facilitate physician annotation and model training. Next, we use a pre-trained classification model to screen out patches with blood vessels. We select a total of 2000 patches containing blood vessels. These patches are annotated by two experienced pathologists (with more than five years of experience in pathology), and then reviewed by expert pathologists (with more than ten years of experience in pathology) after the annotation is completed.

The annotated dataset contains a total of 2000 images, including 4526 blood vessels. They were divided into training set, validation set and test set according to the ratio of 7:2:1. The training set contains 1400 images, including 3445 blood vessels. The validation set consists of 400 images, which include 681 blood vessels. The test set comprises the remaining 200 images, containing 400 blood vessels. All datasets are stored in PNG format. **Table 1** shows the division of the datasets.

**Table 1:** The partitioning of the dataset.

	Name	Proportion	Number of Pictures	Number of Blood vessels
	training set	70%	1400	3445
dataset	validation set	20%	400	681
	test set	10%	200	400
total		100%	2000	4526

#### 3.1.2 ICPR 2012 mitosis detection dataset

The ICPR 2012 mitosis detection dataset was introduced in the ICPR 2012 competition, making it the first publicly available mitosis detection dataset. This dataset consists of five H&E-stained breast cancer biopsy slides. In each slide, a pathologist selects 10 high-power fields (HPF) at 40x magnification, resulting in a total of 50 HPFs in the dataset, which collectively contain more than 300 cells undergoing mitosis. The slides were scanned using various equipment, including an Aperio XT scanner (A scanner), a Hamamatsu Nano Zoomer scanner (H scanner), and a 10-band multispectral microscope. Each HPF’s mitotic cells were annotated by a pathologist. For our experiments, we focused on data obtained from the A scanner, which includes 50 RGB images. Out of these, 35 were allocated for training, and the remaining 15 were designated for testing. Since the image size of 2084 × 2084 pixels isn’t suitable for direct model training, we subdivided it into patches measuring 521 × 521 pixels, resulting in 243 images that contain a total of 323 mitotic cells. Of these, 195 were used for the training set, and the remaining 48 were assigned to the test set.

### 3.2 The proposed PI-YOLO model

**Figure 1:**
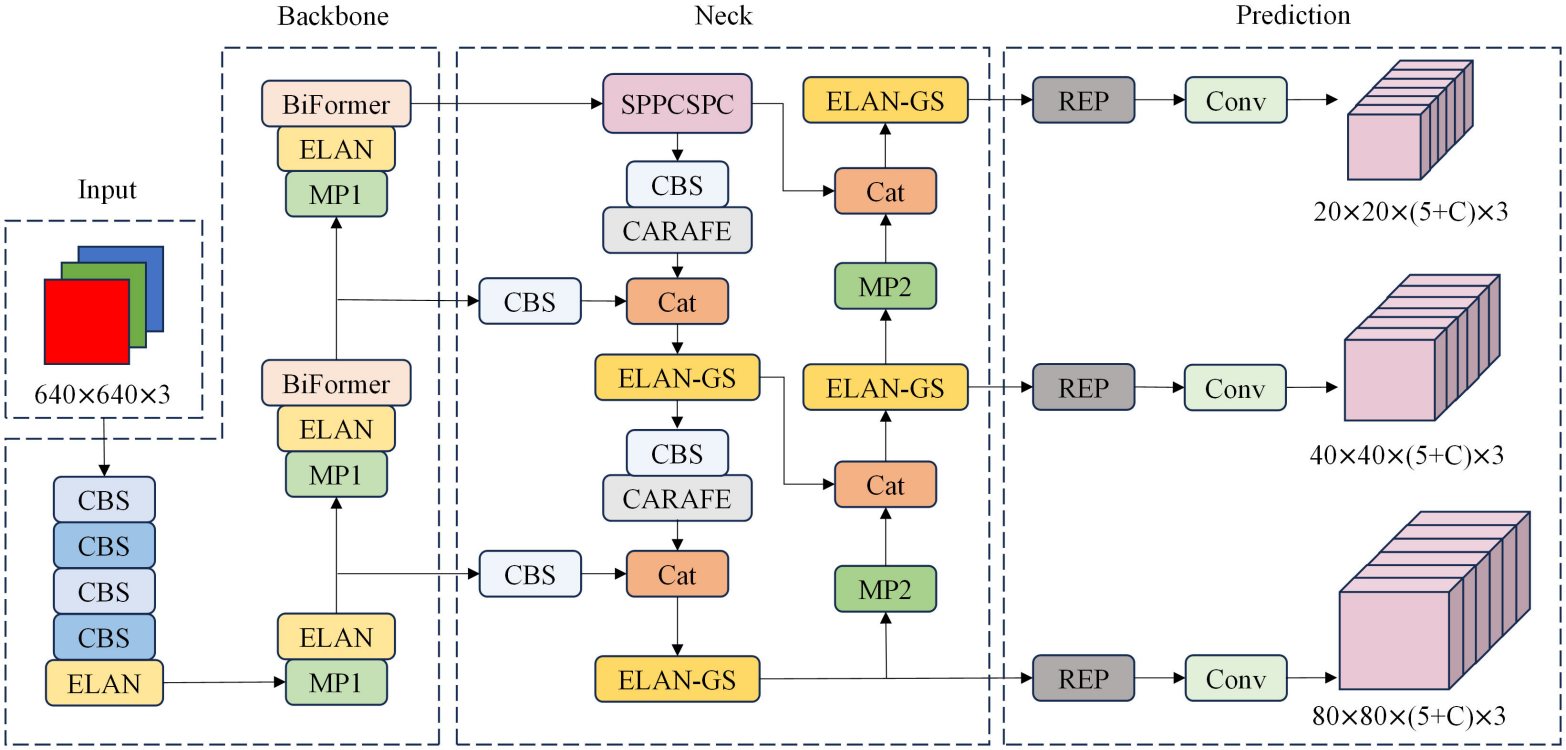
PI-YOLO Network architecture, including Input, Backbone, Neck, and Prediction. C in the Prediction module is the number of categories in the dataset.

Since the background in pathology images typically occupies the majority of the image area, and the target to be detected is often small, it is essential to employ a network structure with global modeling capabilities as the backbone. YOLOv7’s backbone network consists of convolutional stacks. The feature maps in the shallow layers of the network have a limited receptive field, allowing them to capture only local features of the original image. Conversely, the convolutional layers in the deeper network possess a larger receptive field but require more complex computations. BiFormer, a multi-head self-attention mechanism, provides a broader field of view in the shallow layers of the network, enabling it to capture global features. Its key idea lies in filtering out most of the irrelevant key-value pairs at the coarse region level, retaining only a small portion of the routing region. This significantly reduces computational demands while maintaining accuracy. In this paper, we propose a target detection method for pathological images by incorporating the BiFormer attention mechanism, CARAFE upsampling module, GSConv module, and the YOLOv7 network. We have enhanced, optimized, and reconstructed the method. The overall network framework, shown in **Figure 1**, consists of four main components: Input, Backbone, Neck, and Prediction.

#### 3.2.1 Input layer

In the input layer, each training sample undergoes an initial Mosaic data augmentation process. This process involves the following steps: First, four different images are randomly selected from the dataset. Each selected image is then individually flipped, and its color gamut is adjusted. After these adjustments, the images are randomly cropped. Next, these four augmented images are combined into a single new image, forming a new training sample. This Mosaic augmentation technique enriches the background variations and generalizes the features used for detection. Furthermore, the locations of the detection targets in these new composite images are adaptively adjusted according to their original positions in the selected images. By incorporating diverse backgrounds, the model’s ability to detect targets in complex environments is enhanced through training with these augmented samples.

#### 3.2.2 Backbone

**Figure 2:**
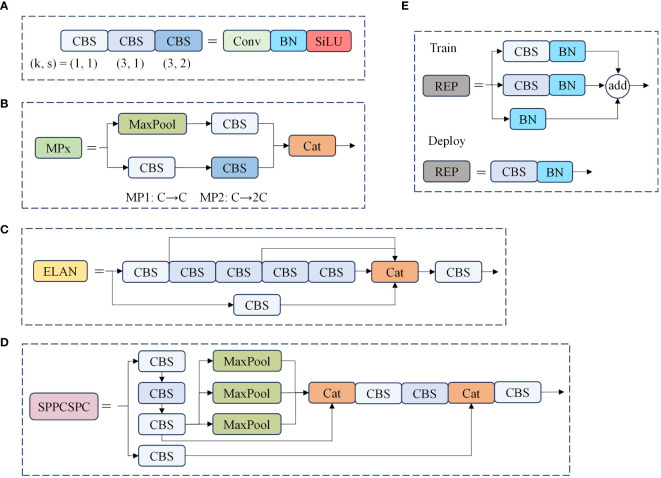
Structure diagram of the model part of the module. In this diagram, **(A)** illustrates the combination of different convolution modules, where “k” represents the convolution kernel size, and “s” signifies the convolution step size. **(B)** outlines the essential configuration of the MP module, while **(C)** provides an overview of the core structure of the ELAN module, **(D)** presents the layout of the SPPCSPC module, and **(E)** describes the architecture of the REP module.

The backbone network is a critical component for feature extraction in our model. The original YOLOv7 backbone consists of 50 modules, which include CBS modules, ELAN modules, and MP1 modules. Specifically, there are four ELAN modules in the network, as shown in **Figure 2**. Each ELAN module is composed of six CBS modules. To enhance the feature extraction capability of the backbone network, we have introduced the BiFormer attention mechanism after the last CBS module of the last two ELAN modules. The BiFormer attention mechanism is characterized by dynamic sparse attention with a two-layer routing process. Its core concept involves filtering out the least relevant key-value pairs at the coarse area level. This process is carried out by constructing and pruning an area-level directed graph. Subsequently, fine-grained token-to-token attention is applied within the union of the routed areas. The incorporation of the BiFormer attention mechanism enables dynamic query-aware sparsity, allowing for more flexible computational allocation and content awareness. This mechanism not only preserves dependencies and location information across different spatial regions but also significantly reduces computational costs. The workflow of the enhanced backbone network can be summarized as follows: Initially, input images pass through a series of CBS modules for basic feature extraction. These features are then fed into the ELAN modules, where the BiFormer attention mechanism is applied to enhance the relevant feature maps. Finally, the refined features proceed to subsequent network layers for further processing and prediction tasks. This structured approach ensures that the backbone network effectively captures and utilizes critical spatial information, ultimately improving the model’s overall performance in object detection tasks.

#### 3.2.3 Neck and prediction layer

The neck network serves to disperse the multi-scale output learned from the backbone network into multiple feature mappings, and then integrates the learned multi-scale information. This is to enhance the model’s ability to capture diverse information and improve target detection performance. As shown in **Figure 1**, the neck network adopts a PAFPN structure, which combines enhanced components from FPN (29) and PANet (30) for feature extraction and fusion. In place of the original upsampling module, we introduce a lightweight generalized upsampling operator called CARAFE within the neck network. This operator expands the receptive field without significantly increasing computational demands or introducing excess parameters. It efficiently leverages semantically relevant content from the feature map for upsampling. Additionally, we employ the lightweight convolutional block GSConv to enhance the ELAN module, reducing model parameters, computational complexity, and size while preserving rich features. Finally, after the input image undergoes two rounds of feature extraction via the backbone and neck networks, the feature information is amalgamated using repconv and transformed into the final prediction information to generate the model’s prediction results.

### 3.3 Attention for PI-YOLO

Due to the intricate backgrounds and a high prevalence of small objects in pathological images, numerous detection models struggle to effectively filter out background information. To shift the focus of the detection model towards the essential information within the input features while minimizing the influence of background data, we incorporate a dynamic sparse attention mechanism known as BiFormer into the backbone network of the model. This BiFormer attention mechanism, as utilized in this study, can be delineated into two distinct phases.

The first phase initiates with coarse-grained attention, emphasizing sparsity control, while the second phase performs fine-grained attention based on the outcomes of the sparse attention from the first phase. In the initial phase, the image is partitioned into multiple coarse-grained blocks, upon which self-attention is applied. This process computes correlations between every two coarse-grained blocks using Q and K , resulting in a relational matrix. Subsequently, this matrix is sparsified, retaining only the top- k elements with the highest values, signifying pairs of blocks that require further attention. In the subsequent phase, building upon the sparse coarse-grained matrix from the first stage, additional fine-grained self-attention is conducted. Each patch exclusively engages in attention computations with patches residing within other coarse-grained blocks that are associated with the coarse-grained block it occupies in the first stage. The implementation details are as follows:

BiFormer is built using Bi-Level Routing Attention (BRA) as the basic building block. The implementation details of BRA are as follows: given a 2D input feature map $X\in R^{H*W*C}$ , it is first divided into S×S non-overlapping regions to obtain Q,K,V , the related equation is as in (1):


(1) $$Q=X^{r}W^{q},\;K=X^{r}W^{k},\;V=X^{r}W^{v}$$

where $W^{q},\;W^{k},\;W^{v}\in R^{c*c}\;$ are projection weights for the query, key, value, respectively.

Then the mean of Q and K is calculated to obtain the corresponding $\;Q^{r}$ , $K^{r}\in R^{S^{2}\times C}$ , and then the affinity adjacency matrix $A^{r}\in R^{S^{2}\times S^{2}}$ between regions is obtained using transpose multiplication, the related equation is as in (2):


(2) $$A^{r}=Q^{r}{\left(K^{r}\right)}^{T}$$

Then use the topk operator to keep the k regions with the closest relationships to obtain the region routing index matrix $I^{r}$ , the related equation is as in (3):


(3) $$I^{r}=topkIndex\left(A^{r}\right)$$

**Figure 3:**
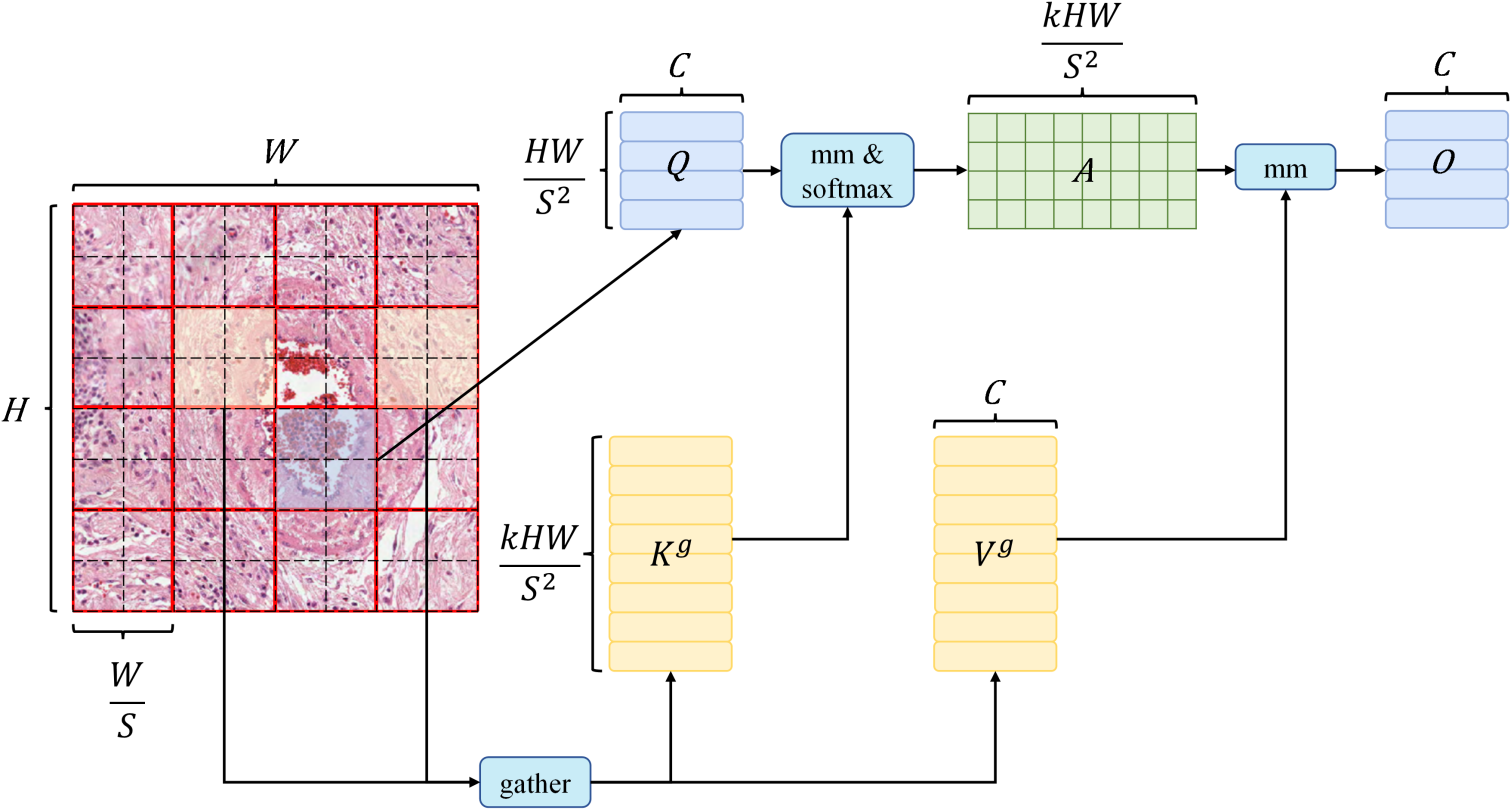
The structure diagram of the BiFormer dynamic attention mechanism.

After obtaining $I^{r}$ , fine-grained Token-to-token attention can be applied, as shown in the **Figure 3**.

First, collect all the routing regions indexed by all elements in $I^{r}$ and collect all their K and V to obtain $K^{ɡ}$ , $V^{ɡ}\in R^{S^{2}\times \frac{kHW}{S^{2}}\times C}$ , the related equation is as in (4):


(4) $$K^{ɡ}=ɡather\left(K,I^{r}\right),\;V^{ɡ}=gather\left(V,I^{r}\right)$$

Then apply $K^{ɡ}$ and $V^{ɡ}$ , which are the gathered key and value tensor. Next, apply attention to the gathered key-value pairs as follows (5):


(5) $$O=Attention\left(Q,K^{ɡ},V^{ɡ}\right)+LCE\left(V\right)$$

**Figure 4:**
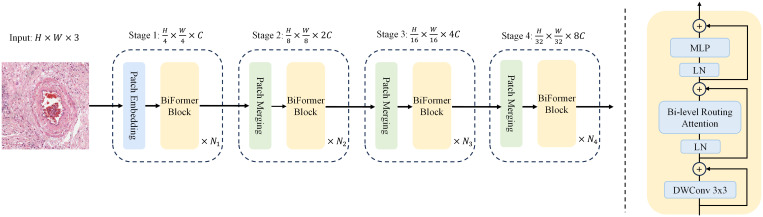
Left: The overall architecture of BiFormer. Right: Details of a BiFormer Block.

Here, a local context enhancement term LCE(V) is introduced, as described in (31). The function LCE(·) is parameterized using deep separable convolution, and set the convolution kernel size to 5. It follows the design of most vision transformer architectures, which also use a four-stage pyramid structure, i.e., downsampling by a factor of 32, as shown in **Figure 4**.

### 3.4 CARAFE for PI-YOLO

The up-sampling method adopted by YOLOv7 in the feature fusion part is nearest neighbor interpolation up-sampling, which determines the up-sampling kernel only by the spatial location of pixel points, and does not utilize the semantic information of the feature map, ignores the possible influence of surrounding feature points, and the perceptual field is small, and the quality of the image after up-sampling is not high. In this paper, the CARAFE lightweight up-sampling operator with a large perceptual field is used to improve the neck, which can make good use of the semantic information of the feature map.

**Figure 5:**
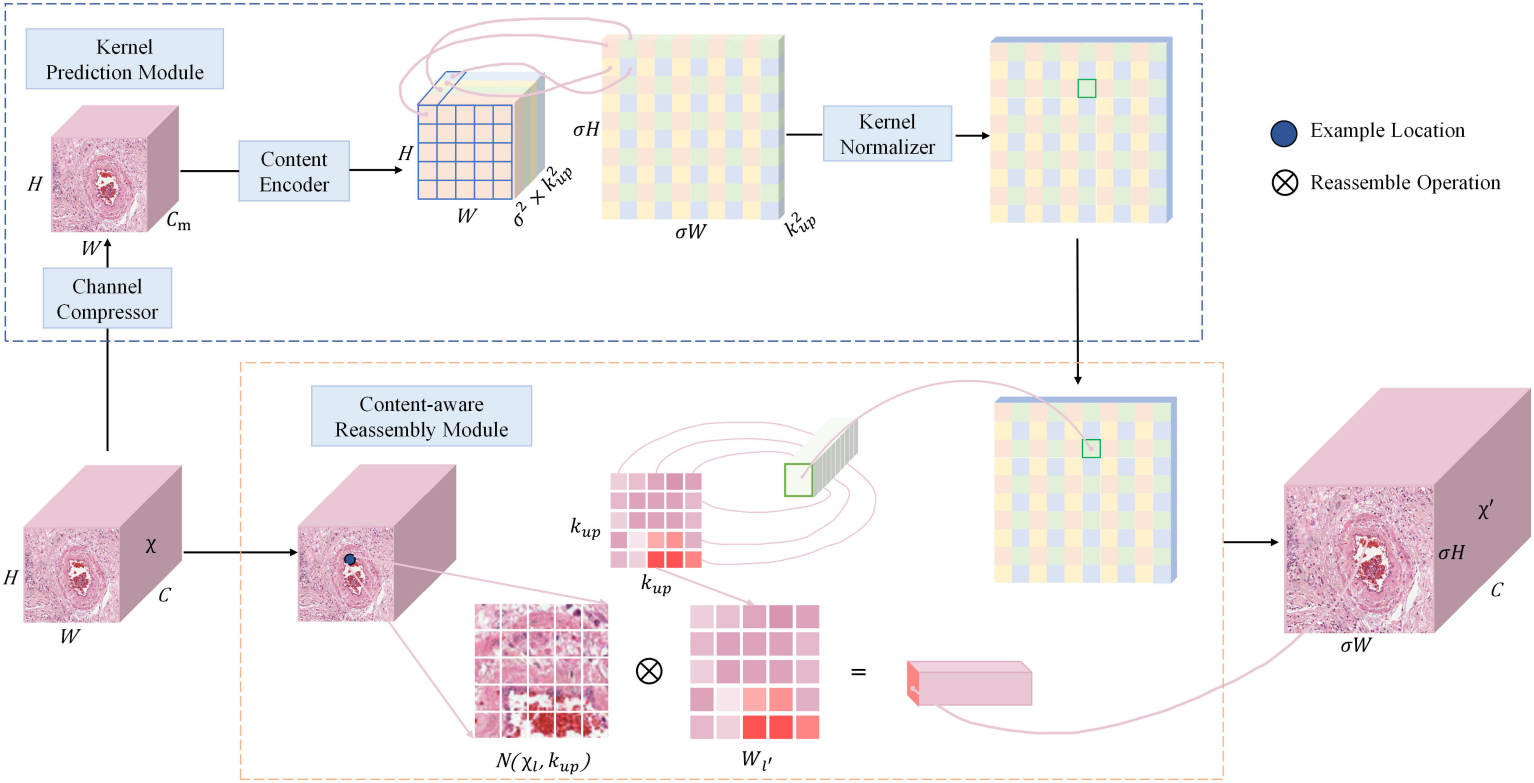
The overall framework of CARAFE. CARAFE is composed of two key components, kernel prediction module and content-aware reassembly module.

CARAFE is divided into a kernel prediction module and a content-aware reassembly module. The kernel prediction module is responsible for generating the up-sampling reassembly kernel, which predicts the attention weights for each up-sampling location based on the mapping relationship between the down-sampled feature map and the up-sampled location. These weights are crucial for maintaining spatial details and contextual information during the feature reassembly process. The content-aware reassembly module focuses on retaining as much spatial information as possible during the up-sampling process to better preserve the accuracy of object boundaries. The structure of CARAFE is shown in **Figure 5**.

The overall sampling process of CARAFE is as follows. Firstly, for the input feature map χ  of shape H×W×C , a 1×1 convolutional layer is used to compress the input channel from C to $C_{m}$ in order to reduce the parameter and computational cost of the model. Next, a reorganization upsampling kernel of shape $H\times W\times C_{m}$ is obtained based on the feature map of shape $H\times W\times \sigma^{2}\times k_{up}^{2}$ by using a convolutional layer as a content encoder for predicting the upsampling kernel, where σ is the upsampling multiplicity and $k_{up}$ is the size of the receptive field region for the feature recombination process. Then the channel is expanded in the spatial dimension to obtain the upsampling kernel of shape $\sigma H\times \sigma W\times k_{up}^{2}$ , and finally the upsampling kernel is normalized so that its convolutional kernel weights sum to 1. In the content-aware reassembly module, for each position in the output feature map, it is mapped back to the input feature map by taking the region centered on the $k_{up}\times k_{up}$ region centered on it, and perform dot product with the predicted upsampling kernel at that point to get the output value. Different channels at the same location share the same upsampling kernel, and finally the σH×σW×C upsampled feature map $\chi {\;}^{'}$ is obtained.

The CARAFE upsampling module enhances the ability of the neck network for image feature extraction and fusion, thus effectively addressing the challenges posed by the presence of a large number of backgrounds and densely distributed small targets in pathology images.

### 3.5 GSConv for PI-YOLO

Standard Convolution (SConv) operates on all three channels simultaneously, where the number of convolution kernels and channels matches the number of output and input channels, respectively. Consequently, employing an excessive number of standard convolution kernels results in an accumulation of parameters. Utilizing SConv for image feature extraction leads to a proliferation of parameters and feature redundancy, particularly in deeper layers. The Ghost Conv model module, proposed by Han K et al. (32), efficiently extracts valuable features while reducing parameters and computational overhead. It operates in two steps: initially involving a limited number of convolutional and linear transformation operations, followed by the integration of feature maps generated from these two operations, which are then output.

**Figure 6:**
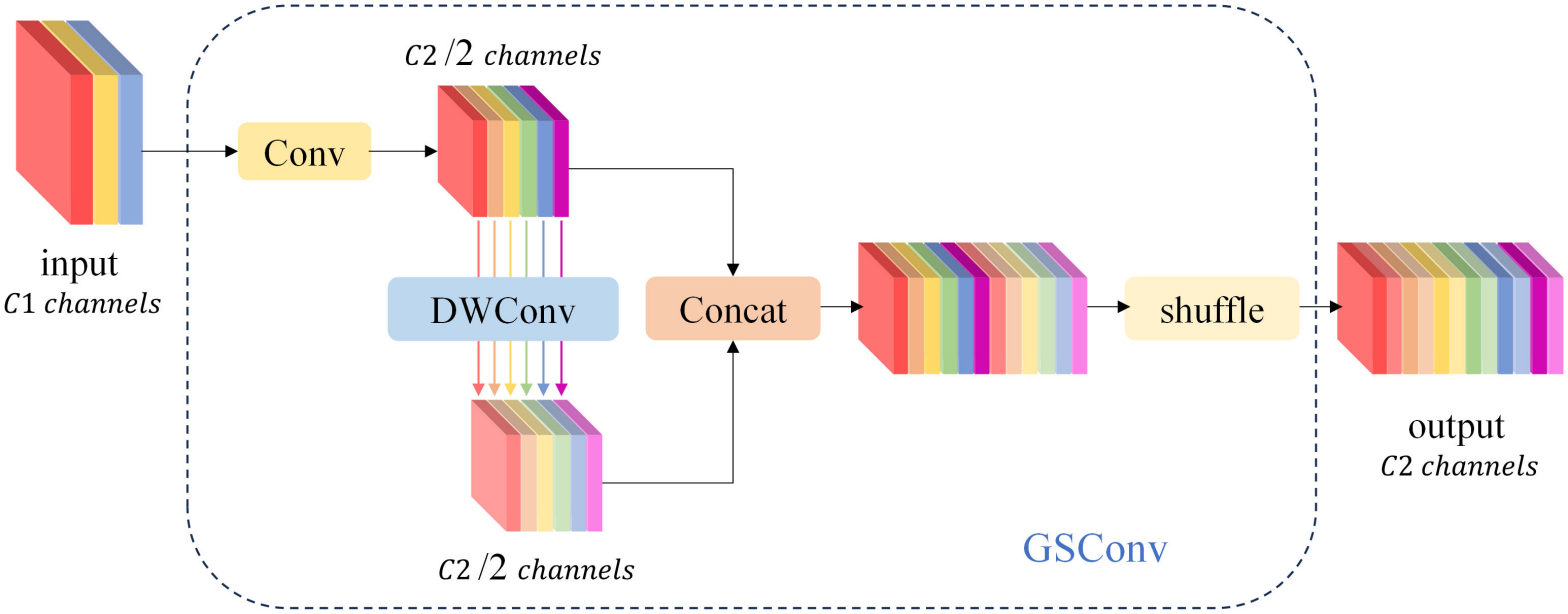
The structure of the GSConv module. The “Conv” box consists of three layers: a convolutional-2D layer, a batch normalization-2D layer, and an activation layer.

Ghost Conv is predominantly employed in the realm of lightweighting computer vision models due to its impressive performance. However, the Ghost Conv module does encounter a challenge in that it loses a significant amount of channel information during its second step of operation. To address this limitation, Li H et al. (16) introduced the GSConv module, as illustrated in **Figure 6**. The GSConv module is designed to mitigate this issue. Its final blending operation effectively disrupts channel information uniformly, enhances semantic information extraction, strengthens the fusion of feature data, and ultimately improves the representation of image features.

**Figure 7:**
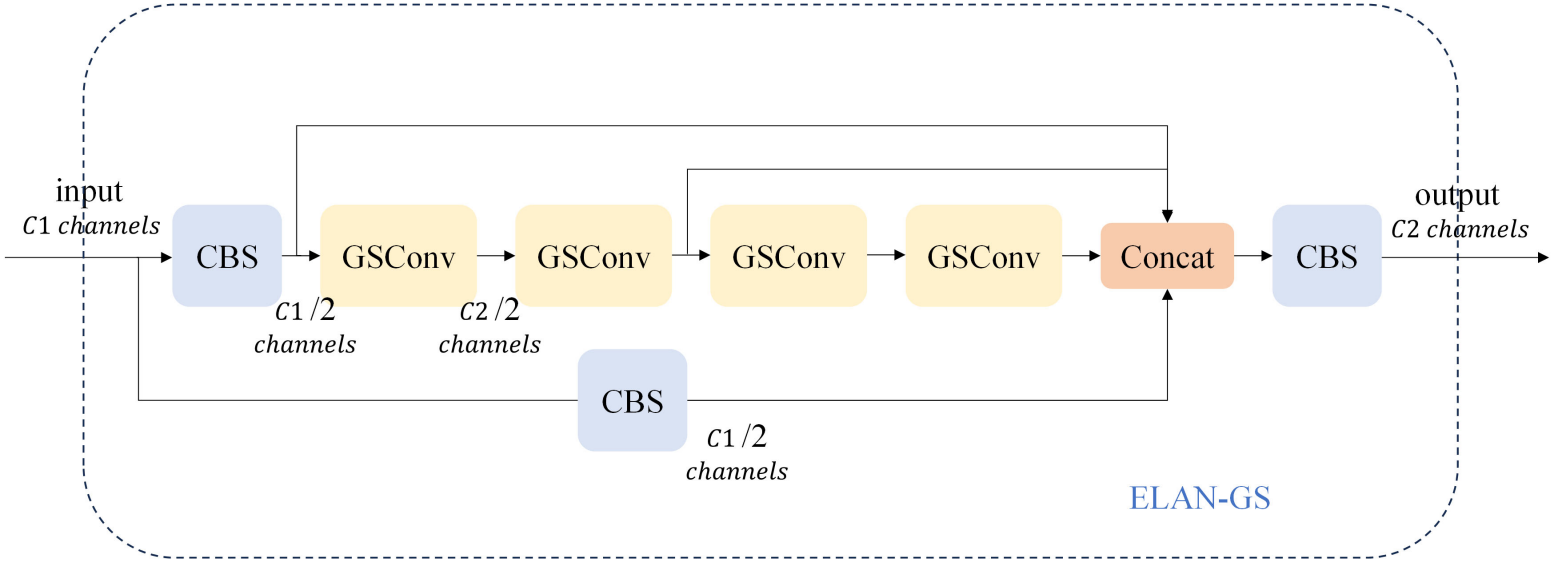
The ELAN-GS structure diagram.

When the network conducts feature fusion at the Neck layer, it continuously propagates semantic information downward. However, this propagation can result in the loss of some semantic information, which may affect the final prediction, especially when the height and width of the feature map, as well as the number of channels, undergo continuous compression and expansion. In this paper, we introduce the GSConv module into the ELAN module of the network’s neck layer, replacing the standard convolution. This adjustment not only reduces the model’s parameter count and computational load but also maximizes the sampling effect. The structure of the GSConv module is illustrated in **Figure 7**. Specifically, the four convolutions preceding the Concat layer make use of the GSConv module. This modification reduces the model’s parameter count while ensuring detection accuracy.

## 4 Experiments and results

### 4.1 Experimental environment and hyperparameter settings

All experimental data in this article were measured in the same environment. The hardware environment adopts Intel (R) Xeon (R) Gold 5218 @ 2.30GHz CPU, 64GB RAM, and NVIDIA GeForce RTX TITAN graphics card. The system environment is Linux version 5.13.0-30 generic. Python version 3.10, PyTorch version 1.13.0, CUDA version 11.7.

In the experimental models presented in this paper, we explored various hyperparameter configurations and found that the best results were obtained when using the default hyperparameters of the original YOLOv7. The relevant parameters used in the experiments are listed in **Table 2**. The gradient descent optimizer employed for updating the convolutional kernel parameters is Adam, with a momentum parameter of 0.937. During the training process, the learning rate is updated using a step-wise method, with a maximum learning rate of 0.001 and a training batch size of 24. The training duration spans 200 epochs, and it’s worth noting that all experiments were conducted without pre-training weights. The entire network model was trained from scratch.

**Table 2:** Experiment-related hyperparameter settings.

Hyperparameter	Epoch	Batch_size	Max_learning_rate	Optimizer	Momentum	Lr decay
Value	200	24	0.001	Adam	0.937	Step

### 4.2 Evaluation indicators

In this study, we utilize seven evaluation metrics to assess the model’s performance:

Precision: This metric represents the ratio of correctly predicted positive instances (TP) to the total recognized objects and is calculated as shown in Equation (6). Recall: Recall signifies the ratio of correctly recognized objects to the total number of objects and is calculated using Equation (7). F1 Score: The F1 score is the harmonic mean of Precision and Recall, computed as indicated in Equation (8). Average Precision (AP): AP is the average of precision values at different recall points, quantified by the area under the Precision-Recall (PR) curve. A higher AP value indicates greater model precision, with the calculation formula shown in Equation (9). Mean Average Precision (mAP): mAP represents the average AP across all categories. A higher mAP value signifies a superior model with increased target recognition accuracy, with the formula outlined in Equation (10). Frames Per Second (FPS): FPS indicates the number of images processed per second and serves as an indicator of detection speed. A higher value implies faster model inference. Giga Floating-point Operations Per Second (GFLOPS): GFLOPS quantifies the computational complexity of the model, reflecting the number of computations required. Additionally, the term “Params” refers to the total number of trainable parameters in the model, serving as an indicator of the model’s size and training requirements.


(6) $$Precision=\frac{TP}{TP+FP}$$

(7) $$Recall=\frac{TP}{TP+FN}$$

(8) $$F1=2\times \frac{Precision\times Recall}{Precision+Recall}$$

(9) $$AP=\int_{0}^{1}P\left(R\right)dR$$

(10) $$mAP=\frac{\sum_{1}^{N}\int_{0}^{1}P\left(R\right)dR}{N}$$

TP represents the count of positive samples correctly predicted by the model, whereas FP  represents the count of negative samples predicted as positive by the model. FN represents the count of positive samples that the model incorrectly predicts as negative. In this context, P represents the class accuracy, R represents the class recall rate, and N represents the total number of classes. Given that the dataset contains only one type of blood vessel, N=1 .

### 4.3 Attention mechanism compatibility experiment

We chose to incorporate the BiFormer attention mechanism into our model. To assess its compatibility with the model, we conducted comparisons with models that lacked a fused attention mechanism, as well as models that integrated the fused SENet, ECA, CA, and CBAM attention mechanisms, respectively (33–36). In our qualitative analysis experiments, we employed a visualization technique commonly used in deep learning, known as Grad-CAM (37), to illustrate differences in the regions of interest within the model after integrating various attention mechanisms. This method offers insights into the model’s focus on different regions and helps explain variations in model performance. The importance of features is depicted using a color scale, with increasing importance denoted by a transition from blue to red hues.

**Figure 8:**
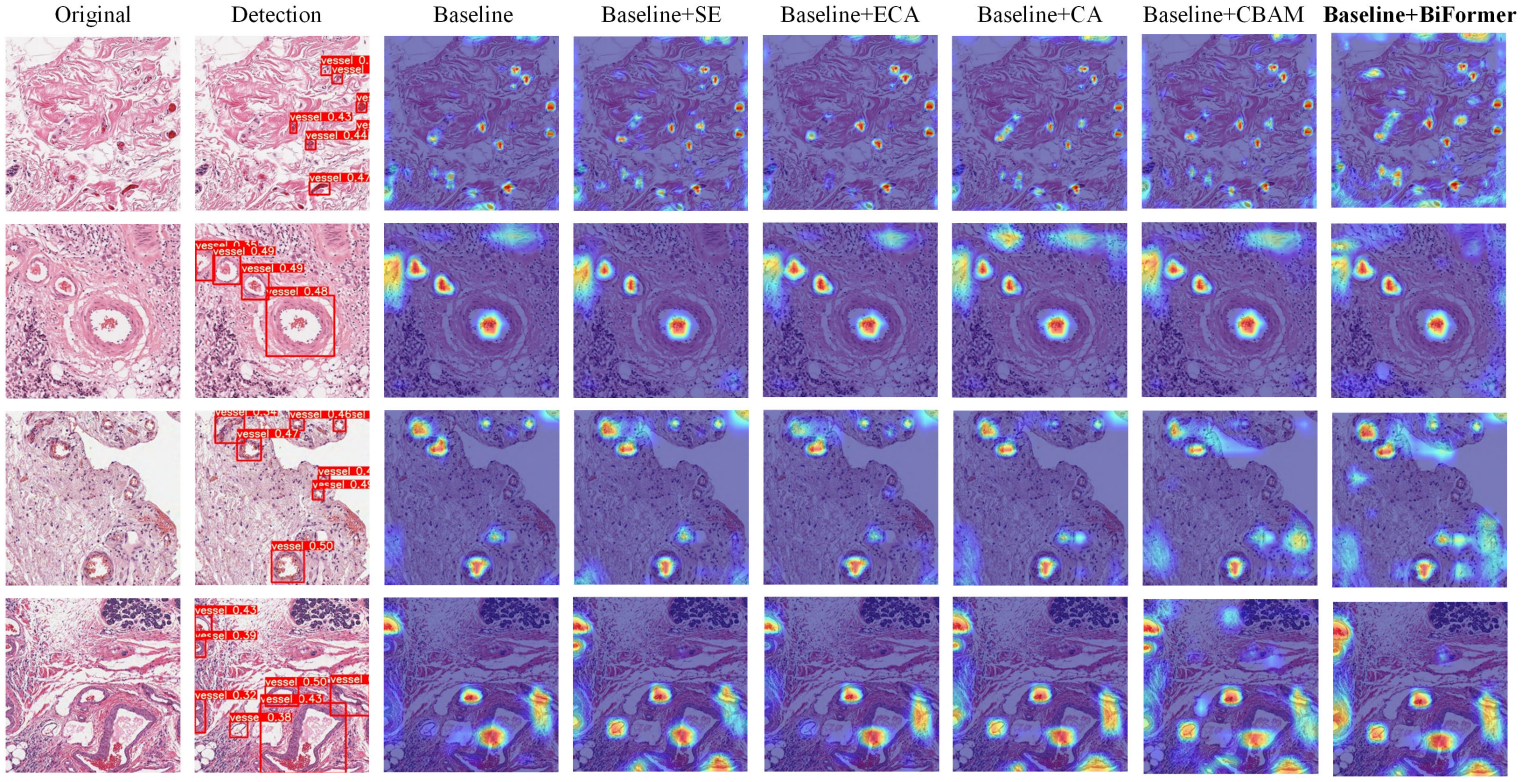
Heatmaps for various attention mechanisms. “Original” displays the dataset image. “Detection” presents the detection results of Baseline+BiFormer, while “Baseline” exhibits the heatmap of YOLOv7. “Baseline+XX” showcases the heatmap of YOLOv7 integrated with the XX attention mechanism (where XX represents SE, ECA, CA, CBAM, and BiFormer).

As illustrated in **Figure 8**. Heatmap, which displays the heatmaps generated by different attention mechanisms, we conducted experiments using images from various sources. When compared with the visualization results of other attention mechanisms, it becomes evident that the heatmap produced by the BiFormer Attention Mechanism exhibits a larger overall coverage area. This suggests that the model focuses on a broader region of interest at the target location, resulting in more comprehensive feature extraction of the targets. This, in turn, facilitates the detection of small targets. Additionally, the red area in the heatmap is also more extensive, indicating enhanced extraction of effective target feature information. The model allocates greater attention to the pertinent target information. The experimental outcomes reveal that the integration of the BiFormer attention mechanism compels the model to prioritize the feature information of the target to be recognized. It also suppresses the influence of target features that may be less conspicuous due to the complexity of the background in pathology images. In comparison with other attention mechanisms, the BiFormer mechanism exhibits superior performance.

We performed a quantitative analysis of the experiment using the mAP evaluation criterion. We introduced changes only to the attention mechanism module, subsequently measuring the mAP values for each model. This allowed us to compare the mAP values among different models, assessing the compatibility between various attention mechanisms and the models. The comparative experimental data is presented in **Table 3**. The results indicate that the model equipped with the BiFormer attention mechanism achieved higher detection accuracy compared to the model without any attention mechanism, as well as models incorporating the SE, ECA, CA, and CBAM attention mechanisms. Specifically, the model incorporating the ECA attention mechanism experienced a 0.28% reduction in detection accuracy. On the other hand, the models incorporating the SE attention mechanism, CA attention mechanism, and CBAM attention mechanism demonstrated improvements in detection accuracy by 0.56%, 1.02%, and 0.09%, respectively. Notably, the model’s detection accuracy was enhanced by 1.48% with the inclusion of the fused BiFormer attention mechanism. These results indicate that, in comparison with the original YOLOv7 model, our model is better suited for handling pathology images.

**Table 3:** mAP measurements for different attention mechanisms.

Model	Attention	Input shape	mAP(%)
YOLOv7	–	640×640	84.65
YOLOv7	SENet	640×640	85.21
YOLOv7	ECA	640×640	84.37
YOLOv7	CA	640×640	85.67
YOLOv7	CBAM	640×640	84.74
YOLOv7	BiFormer	640×640	86.13

### 4.4 Ablation experiment

This portion of the experiment investigates the impacts of the three improvement methods on the network model. The plotted data is presented in **Table 4**. We conducted eight sets of experiments with different modules added, and compared them with the original YOLOv7 model using metrics such as mAP, F1, Params, and FPS. For clarity and convenience, we have designated the network with the BiFormer attention module as “YOLOv7+BiFormer”, the network with the CARAFE upsampling module as “YOLOv7+CARAFE”, and the network with the GSConv convolution as “YOLOv7+GSConv”, and so forth.

**Table 4:** The impact of the fusion of different modules of the model on the metrics.

Methods	mAP(%)	F1(%)	Params(M)	FPS
YOLOv7	84.65	82.45	36.48	64.93
YOLOv7+BiFormer	86.13	83.36	37.01	60.67
YOLOv7+ CARAFE	85.21	82.48	36.72	73.52
YOLOv7+ GSConv	85.59	83.12	33.80	66.89
YOLOv7+ BiFormer+ CARAFE	84.12	81.69	37.26	64.88
YOLOv7+ BiFormer+ GSConv	86.48	83.45	34.33	61.35
YOLOv7+ CARAFE+ GSConv	85.64	81.67	34.45	68.49
YOLOv7+BiFormer+ CARAFE+ GSConv	87.48	85.18	34.90	65.39

As shown in **Table 4**, the incorporation of the BiFormer attention module, CARAFE upsampling module, and GSConv convolution into YOLOv7 leads to a slight improvement in the network’s detection accuracy. Specifically, these improvements are 1.48%, 0.56%, and 0.94% higher than the YOLOv7 model, respectively. This suggests that the integration of the BiFormer attention module directs the model’s attention more effectively toward the feature information of the detection target, enhancing the quality of feature mapping and significantly improving overall accuracy. However, it’s worth noting that the BiFormer attention mechanism increases the model’s complexity and reduces network inference speed. Additionally, we observed that the model’s inference speed can be substantially increased to 73.52 FPS after incorporating the CARAFE upsampling module into the network, which represents a 13.2% improvement over the original version. Furthermore, the fusion of the GSConv convolution module results in a reduction of the model’s parameters to 33.80M, a 7.3% decrease compared to the original version.

Moreover, when combining these modules in pairs, it becomes evident from the table that the combination of BiFormer + GSConv modules exhibits the most substantial improvement in model accuracy. The combination of CARAFE + GSConv modules enhances the model’s inference speed to 68.49 FPS. It is important to note that the introduction of the BiFormer attention module increases both the number of parameters and the inference time of the model. Nevertheless, we assert that this combination of three modules is well-suited. By leveraging the CARAFE module and GSConv module for accelerated inference and lightweight deployment, the incorporation of the BiFormer attention module yields a qualitative improvement in detection accuracy. In summary, our approach demonstrates improvements in both detection accuracy and speed compared to the original model, marking a significant enhancement.

### 4.5 Comparative experiments with other mainstream algorithms

Our proposed PI-YOLO algorithm demonstrates strong feature extraction capabilities in complex pathological image scenes and achieves fast detection speeds, making it a high-performing solution for pathological image object detection. To validate the superiority of our proposed algorithm in the context of pathological images, we conducted comparisons with mainstream object detection algorithms, including Faster RCNN (38), SSD (39), RetinaNet (19), YOLOv5 (40), and YOLOv7 (13). Utilizing the same vascular dataset and training methodology, we performed both qualitative and quantitative analyses to assess the respective advantages of these algorithm models.

**Figure 9:**
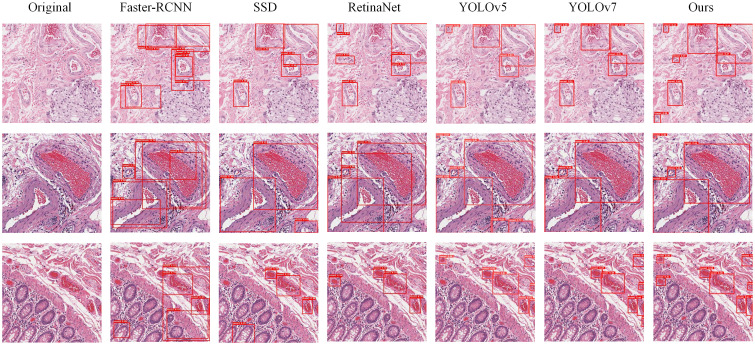
Images displaying the detection results of six models. The first column, “Original,” represents the original images from the dataset. The second column shows the detection images from Faster-RCNN, the third column from SSD, the fourth column from RetinaNet, the fifth column from YOLOv5, the sixth column from YOLOv7, and the seventh column from PI-YOLO.

In our experiments, we conducted a qualitative analysis of the algorithms’ performance by examining the detection result plots of different models. **Figure 9** displays the detection results of Faster-RCNN, SSD, RetinaNet, YOLOv5, YOLOv7, and PI-YOLO. From the visual results, it becomes evident that RetinaNet’s detection performance is superior to that of Faster-RCNN and SSD, and it is on par with YOLOv5. However, the number of detected targets in RetinaNet is generally lower than that in YOLOv5, and there are instances of target misclassifications. The PI-YOLO algorithm introduced in this paper exhibits better target recognition capabilities compared to Faster-RCNN, SSD, and RetinaNet. It also demonstrates fewer misclassifications and identifies a greater number of small vessels compared to YOLOv5.

**Figure 10:**
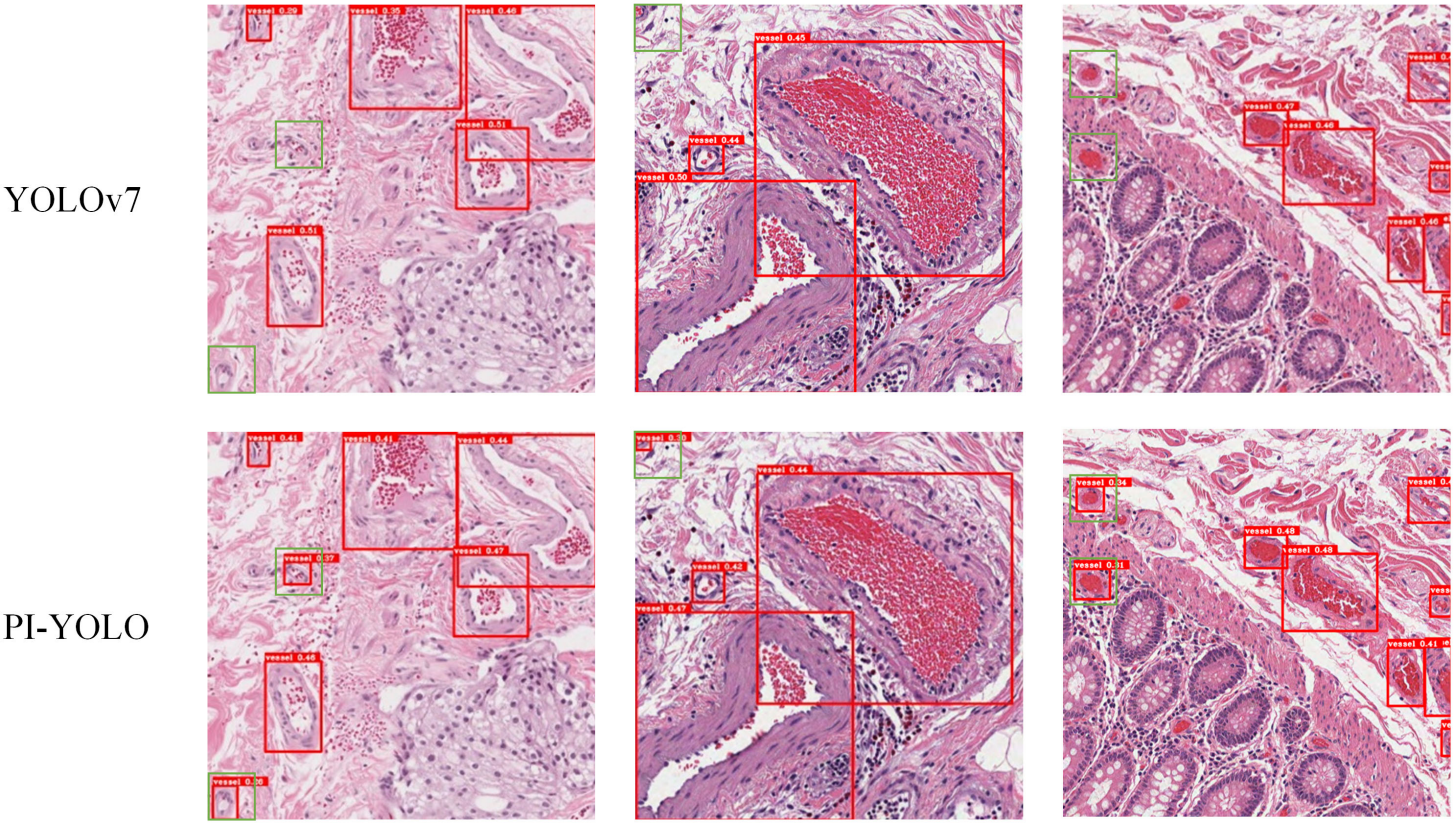
The detection effect of YOLOv7 and the detection effect of PI-YOLO.

To facilitate a comprehensive evaluation of the detection performance of the PI-YOLO algorithm, we conducted a comparative analysis between PI-YOLO and the leading detection algorithm in the field, YOLOv7. **Figure 10**. presents the detection results of both YOLOv7 and PI-YOLO on pathological sample images featuring small blood vessels with unclear edges. These vessels are highlighted with green bounding boxes. As observed, due to the indistinct edges of the small vessels, YOLOv7 struggles to distinguish them from the background, resulting in missed detections and false negatives. In contrast, PI-YOLO accurately identifies and delineates these small vessels. This improvement is attributed to the integration of the BiFormer attention mechanism, which enhances feature extraction, particularly for small and inconspicuous targets.

In our experimental assessment, we quantitatively compared and analyzed each model, employing metrics such as mAP, F1 score, Params, GFLOPS, and FPS. The outcomes of these comparative measurements are presented in **Table 5**. The data indicate that our PI-YOLO algorithm achieves an mAP of 87.48%, surpassing currently mainstream object detection algorithms. In terms of detection speed, the integration of the CARAFE up-sampling module and the GSConv module has effectively reduced the model’s parameter count and increased computational speed, thereby maintaining commendable real-time performance. Notably, our enhanced PI-YOLO algorithm exhibits a significant improvement in accuracy by 18.94% and performance by 90.97% compared to the widely used two-stage object detection algorithm, Faster-RCNN-ResNet. In contrast, when compared with the commonly adopted single-stage object detection algorithm YOLOv5, our PI-YOLO algorithm shows a 4.79% increase in mAP, although the detection speed is slightly reduced by 2.91%. Additionally, compared to the YOLOv7 algorithm, our improved PI-YOLO algorithm shows an increase of 2.83% in mAP and a modest increase of 0.71% in detection speed, while the model size has been reduced by 1.58 M.

**Table 5:** Performance metric values of mainstream target detection algorithms on the dataset.

Model	mAP(%)	F1(%)	FPS	GFLOPS(G)	params(M)
Faster-RCNN-ResNet	68.54	64.37	34.24	416.52	127.35
SSD	75.23	73.36	107.64	215.37	23.72
RetinaNet	80.34	74.47	44.69	120.43	35.56
YOLOv5	82.69	80.15	67.35	115.32	45.53
YOLOv7	84.65	82.45	64.93	103.23	36.48
PI-YOLO	87.48	85.18	65.39	119.70	34.90

### 4.6 Comparative experiments on other detection tasks in pathological images

**Figure 11:**
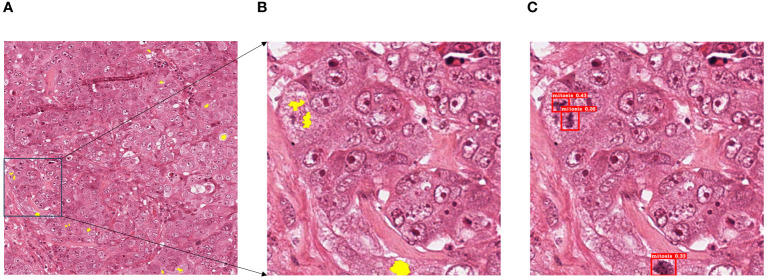
Schematic diagram of mitotic assay results, **(A)** ground truth; **(B)** patch of ground truth; **(C)** test results.

To demonstrate the superior performance of the PI-YOLO algorithm in pathology image detection, we conducted experiments using the ICPR 2012 mitotic target detection dataset. The experimental results are presented in **Figure 11**. We measured the Precision, Recall, and F1 values of the model and compared them with the current state-of-the-art mitosis detection methods using different metrics. The comparison results are summarized in **Table 6**. While PI-YOLO falls within the middle range in terms of Precision, it achieves the highest Recall value among all methods, leading to the highest F1 score as well. These results indicate that the PI-YOLO algorithm excels in feature extraction, particularly in the context of complex pathology images from various sources and tissues.

**Table 6:** Performance index values of different methods on the ICPR 2012 mitosis detection dataset.

Methods	Dataset	Precision	Recall	F1
H. Chen et al. (41)	MITOS-12-scanner A	0.8040	0.7720	0.7880
M. Ma et al. (42)	MITOS-12-scanner A	0.7760	0.7870	0.7810
C. Li et al. (43)	MITOS-12-scanner A	0.8540	0.8120	0.8320
H. Lei et al. (44)	MITOS-12-scanner A	0.9200	0.7920	0.8510
T. Mahmood et al. (45)	MITOS-12-scanner A	0.8760	0.8410	0.8580
M. Sebai et al. (46)	MITOS-12-scanner A	0.9210	0.8110	0.8630
ours	MITOS-12-scanner A	0.8589	0.8769	0.8678

In summary, the proposed model achieves the highest detection accuracy among current mainstream detection algorithms and also maintains good detection and inference speed. The network demonstrates significant advantages in pathology image object detection tasks.

## 5 Conclusion

In this paper, we introduce the PI-YOLO target detection model to achieve automated blood vessel detection in pathology images using deep learning techniques. Our research addresses the challenges presented by pathology images, which include a high proportion of small targets, complex image backgrounds, dense target distribution, and subtle feature differences between the target and the background. Our model incorporates the BiFormer attention mechanism, which effectively reduces information loss during feature extraction while capturing long-range contextual dependencies. This not only saves computational resources but also enhances the overall feature extraction capabilities of the network. The integration of this attention mechanism into YOLOv7 results in improved detection accuracy for pathology images. Furthermore, by replacing the upsampling module and implementing GSConv convolution, we maintain detection accuracy while reducing model parameters and enhancing inference speed. These components, when integrated into YOLOv7, yield the enhanced PI-YOLO model. This model demonstrates superior performance in pathology image detection tasks, achieving a remarkable mAP value of 87.48%. It partially mitigates the challenges posed by complex backgrounds in pathology images. Moreover, automating blood vessel detection in pathology images significantly assists researchers in the study of anti-tumor vascular therapy, offering substantial medical value.

However, although our method is highly effective in vascular detection tasks, it currently lacks the capability to differentiate among various types of blood vessels, such as arterial, venous, and capillary. This limitation affects its specificity in tumor studies where such distinctions are crucial. Deploying PI-YOLO in clinical settings presents several challenges, including the need for high computational resources, seamless integration into existing diagnostic workflows without disruption, and robustness against variability in pathology image data due to differing laboratory standards and imaging equipment. To overcome these challenges and improve the model, our future research will focus on developing methods to accurately distinguish between different blood vessel types to enhance clinical relevance in tumor analysis. We aim to optimize the detection speed of our models for real-time clinical use and expand our dataset to include a more diverse range of pathology images, thereby improving the model’s generalizability and robustness. Additionally, we plan to explore deployment on embedded devices to provide on-site assistance to medical professionals, facilitating quicker and more accurate diagnosis and treatment decisions. These steps will pave the way for the successful implementation of PI-YOLO in practical medical applications, ultimately benefiting patient care.

## Data availability statement

Publicly available datasets were analyzed in this study. This data can be found here: https://portal.gdc.cancer.gov/ and https://ipal.cnrs.fr/ICPR2012/. The private dataset is not available because of privacy regulations.

## Ethics statement

Ethical approval was not required for the studies on humans in accordance with the local legislation and institutional requirements because only commercially available established cell lines were used.

## Author contributions

CL: Conceptualization, Data curation, Formal analysis, Investigation, Methodology, Project administration, Software, Supervision, Validation, Visualization, Writing – original draft, Writing – review & editing. SC: Data curation, Resources, Validation, Writing – review & editing. HG: Investigation, Software, Visualization, Writing – review & editing. YD: Software, Writing – review & editing. YL: Writing – review & editing. JX: Writing – original draft, Writing – review & editing. LQ: Data curation, Writing – review & editing. GZ: Funding acquisition, Methodology, Project administration, Resources, Writing – review & editing.
