# Corrigendum: PI-YOLO: dynamic sparse attention and lightweight convolutional based YOLO for vessel detection in pathological images

**DOI:** 10.3389/fonc.2024.1472621

**Published:** 2024-08-09

**Authors:** Cong Li, Shuanlong Che, Haotian Gong, Youde Ding, Yizhou Luo, Jianing Xi, Ling Qi, Guiying Zhang

**Affiliations:** ^1^ Affiliated Qingyuan Hospital, The Sixth Clinical Medical School, Guangzhou Medical University, Qingyuan People’s Hospital, Qingyuan, Guangdong, China; ^2^ Department of Pathology, Guangzhou KingMed Center for Clinical Laboratory, Guangzhou, China; ^3^ School of Health Management, Guangzhou Medical University, Guangzhou, China; ^4^ School of Biomedical Engineering, Guangzhou Medical University, Guangzhou, China; ^5^ Institute of Digestive Disease, the Sixth Affiliated Hospital of Guangzhou Medical University, Qingyuan People’s Hospital, Qingyuan, China

**Keywords:** pathological images, blood vessel, deep learning, object detection, attention mechanism

In the published article, there was an error in affiliation 1. Instead of “School of Biomedical Engineering, The Sixth Affiliated Hospital of Guangzhou Medical University, Qingyuan People’s Hospital, Guangzhou Medical University, Guangzhou, China”, it should be “Affiliated Qingyuan Hospital, The Sixth Clinical Medical School, Guangzhou Medical University, Qingyuan People’s Hospital, Qingyuan, Guangdong, China”.

The authors apologize for this error and state that this does not change the scientific conclusions of the article in any way. The original article has been updated.

