# Corrigendum: PI-YOLO: dynamic sparse attention and lightweight convolutional based YOLO for vessel detection in pathological images

**DOI:** 10.3389/fonc.2024.1478598

**Published:** 2024-08-23

**Authors:** Cong Li, Shuanlong Che, Haotian Gong, Youde Ding, Yizhou Luo, Jianing Xi, Ling Qi, Guiying Zhang

**Affiliations:** ^1^ The Affiliated Qingyuan Hospital (Qingyuan Peoples’s Hospital), Guangzhou Medical University, Qingyuan, China; ^2^ School of Biomedical Engineering, Guangzhou Medical University, Guangzhou, China; ^3^ Department of Pathology, Guangzhou KingMed Center for Clinical Laboratory, Guangzhou, China; ^4^ School of Health Management, Guangzhou Medical University, Guangzhou, China; ^5^ Division of Gastroenterology, Institute of Digestive Disease, the Affiliated Qingyuan Hospital (Qingyuan Peoples’s Hospital), Guangzhou Medical University, Qingyuan, China

**Keywords:** pathological images, blood vessel, deep learning, object detection, attention mechanism

In the published article, there was an error in affiliation 1. Instead of “Affiliated Qingyuan Hospital, The Sixth Clinical Medical School, Guangzhou Medical University, Qingyuan People’s Hospital, Qingyuan, Guangdong, China”, it should be “the Affiliated Qingyuan Hospital (Qingyuan Peoples’s Hospital), Guangzhou Medical University, Qingyuan, China”.

In the published article, there was an error in affiliation 5. Instead of “Institute of Digestive Disease, the Sixth Affiliated Hospital of Guangzhou Medical University, Qingyuan People’s Hospital, Qingyuan, China”, it should be “Division of Gastroenterology, Institute of Digestive Disease, the Affiliated Qingyuan Hospital (Qingyuan Peoples’s Hospital), Guangzhou Medical University, Qingyuan, China”.

In the published article, there was an error regarding the affiliations for Cong Li. As well as having affiliation 1, they should also have 2.

In the published article, there was an error regarding the affiliations for Ling Qi. As well as having affiliation 5, they should also have 1.

In the published article, there was an error regarding the affiliations for Guiying Zhang. As well as having affiliation 1, they should also have 2.

The authors apologize for these errors and state that this does not change the scientific conclusions of the article in any way. The original article has been updated.

